# From Covalent Systems to Bulk Phases: Addressing Structural Complexity with Computational NMR

**DOI:** 10.1021/acs.accounts.6c00045

**Published:** 2026-03-10

**Authors:** Giacomo Saielli

**Affiliations:** CNR Institute on Membrane Technology “Enrico Drioli”, Unit of Padova, Via Marzolo, 1, 35131 Padova, Italy; Department of Chemical Sciences, University of Padova, Via Marzolo, 1, 35131 Padova, Italy

## Abstract

The free induction decay (FID) signal acquired in a typical NMR experiment contains information about the chemical shifts, δ, and the spin–spin coupling constants, *J*, of the system investigated. These two parameters, particularly the chemical shifts, are very sensitive to both intramolecular and intermolecular perturbations. As such, they are also very good probes of the structure of both the molecule and the hosting solvent/matrix. This sensitivity is exploited in natural product studies to deduce the molecular structure of newly isolated compounds from the analysis of their NMR spectra. However, for complex carbon skeletons, the interpretation of the NMR data is far from trivial; the structural information is too deeply buried within the overlapping high-order multiplets and close resonances. In these cases, it is useful to compare the experimental NMR data of the unknown substance with the ones predicted by density functional theory (DFT) based methods for hypothetical molecules. Ideally, one will discard all putative structures resulting in a disagreement with the experiments and will keep the only one exhibiting an agreement within the benchmarked accuracy of the level of theory used. Two other sources of complexity, besides the topological complexity of natural substances, may strongly affect the interpretation of the NMR spectra. One, still related with covalent compounds, is the presence of heavy atoms which brings in relativistic effects in the NMR. Even for a simple molecular structure, they turn the interpretation of the NMR spectrum into a very difficult task since empirical rules often do not allow a full elucidation of the structure; thus, such effects can be accounted for only with relativistic versions of DFT. The other source of complexity is the presence of strong noncovalent interactions of the NMR probe molecule with its environment. In these cases, the full dynamics of the solute and solvent system has to be taken into account and the structure that is responsible for the observed NMR is in fact the average bulk structure of the solute–solvent system. Then, molecular dynamics (MD) simulations have to be coupled with the DFT-NMR calculations in order to predict the NMR properties. In turn, the comparison between the calculated and experimental data can shed light on the force field (FF) parameters used in the MD simulation. Therefore, computational NMR can be used to shed light on both covalent and noncovalent structural problems: in one case, the exploration of a discrete structural space will allow one to select the correct structure of an unknown compound among several hypothesis; in the other one, it will enable the fine-tuning of classical FF parameters over a continuum range of possibilities.

## Graphical abstract

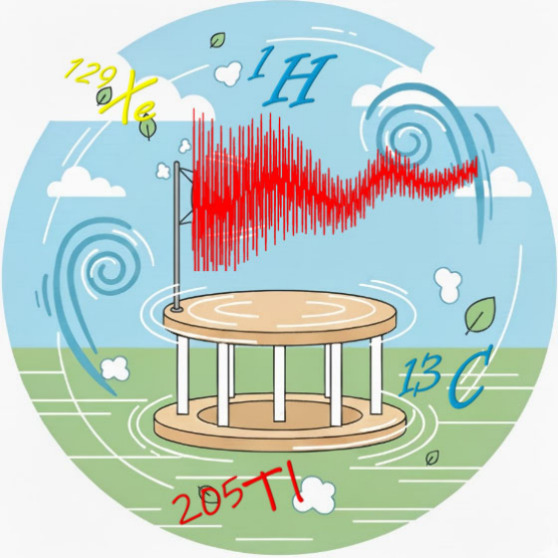

## Key References

- Saielli, G. Computational NMR spectroscopy of ^205^Tl. J. Comput. Chem. 2023, 44, 2016–2029.37367222 10.1002/jcc.27176 ^50^ *Relativistic DFT-NMR calculations of δ­(^205^Tl) highlight some discrepancies in X-ray geometries of thallium compounds.*
- Boventi, M.; Mazzilli, V.; Simonutti, R.; Castiglione, F.; Saielli, G. Exploring the structure of halomethanes with xenon: an NMR and MD investigation. J. Mol. Liq. 2023, 382, 122011.^65^ *DFT-NMR and MD simulations are combined to gain detailed information on the microscopic solvation structure of xenon in dihalomethanes by comparison of calculated and experimental δ­(^129^Xe).*
- Mazzilli, V.; Rizzuto, C.; Tocci, E.; Saielli, G. 
^129^Xe NMR in polymeric membranes: a computational study of the effect of pore size and void distribution on the xenon chemical shift. J. Phys. Chem. B 2025, 129, 11090–11099.40973031 10.1021/acs.jpcb.5c05500PMC12557378 ^66^ *A detailed DFT-NMR analysis of δ­(^129^Xe) in microporous polymeric membranes allows one to derive a general law for the dependence of the xenon chemical shift on the distance from the pore walls.*
- Zhu, R.; Yan, T.; Wang, Y.; Saielli, G. Towards quantitative prediction of proton chemical shifts in imidazolium chloride ionic liquids by computational NMR. Phys. Chem. Chem. Phys. 2025, 27, 25310–25321.41171003 10.1039/d5cp03322e^9^ *Comparison of experimental and calculated δ­(^1^H) of butylmethylimidazolium cation in tetrafluoroborate and chloride salts allows one to pinpoint key features of the MD classical force fields that need to be improved.*

## 1 Introduction

NMR in chemistry is mostly related to the measurement of chemical shifts, δ, and spin–spin coupling constants, *J*, of active atomic nuclei, notably ^1^H and ^13^C for organic molecules, as well as virtually any other nucleus of the periodic table, down to the least sensitive one, ^187^Os, for organometallic and inorganic systems. The chemical shift is defined as

$$\delta =\frac{\sigma_{ref}-\sigma }{1-\sigma_{ref}}\approx \sigma_{ref}-\sigma$$ 1

where σ is the shielding constant of our nucleus of interest and σ_ref_ is the shielding constant of a reference compound (tetramethylsilane, TMS, for ^1^H and ^13^C). Chemical shifts and coupling constants can be extracted directly from one-dimensional spectra, either by visual inspection for first-order spin systems or after simulation for higher order cases. These parameters are purely electronic properties that can also be calculated by means of quantum mechanical (QM) methods, mostly rooted in density functional theory (DFT), as second derivatives of the electronic energy with respect to the external magnetic field or the nuclear magnetic dipole moments.^1^ Once the shielding constant σ is obtained, eq 1 gives the chemical shift δ after calculating the shielding constant of the reference compound using exactly the same level of theory.

The shieldings/chemical shifts, and to a lesser extent the coupling constants, are strongly affected not only by the covalent structure of the molecule of interest but also by the bulk average structure of the environment.^2^

The relationship between the structure of a molecule made of light atoms and its NMR spectra is well understood. Using empirical concepts like inductive and mesomeric effects, Karplus type relationships, Shoolery’s additivity rules, etc., we can easily interpret the NMR spectra of relatively simple organic molecules, a topic often covered in courses like Spectroscopy Methods in Organic Chemistry in several chemistry degrees.^3^ Then, it is possible to infer the structure of an unknown molecule from the set of ^1^H and ^13^C chemical shifts and spin–spin coupling constant. The information flow is therefore from the experimental data into the chemical structure: information really means that the set of δ and *J* enables the chemical structure to take form. This is the well-known field of structural elucidation of organic molecules.

There are, however, some instances where this body of empirical rules may fail and the flow of information mentioned above may be interrupted: (i) Organic molecules made of light atoms but having a complex topology, typically natural products. In this respect, the review paper by Nicolaou and Snyder concerning “Molecules That Were Never There” is emblematic;^4^ (ii) molecules with relatively simple atomic structure, but including heavy elements, having a complex electronic structure due to relativistic effects. A paradigmatic example is the very recent revision of the structure of a octahedral hydride iridium complex with pyruvate and 1,3-dimesitylimidazol-2-yildene, which, despite its structural simplicity, defied structural elucidation until now because of the large relativistic effects on the proton resonances;^5^ (iii) systems with strong interactions between the reference molecule and the environment (solvent, hosting matrix).^2,6^ Of course, all mixed situations can also occur, multiplying the efforts needed to understand the NMR spectra. This situation is schematically represented in Figure 1.

**Figure 1:**
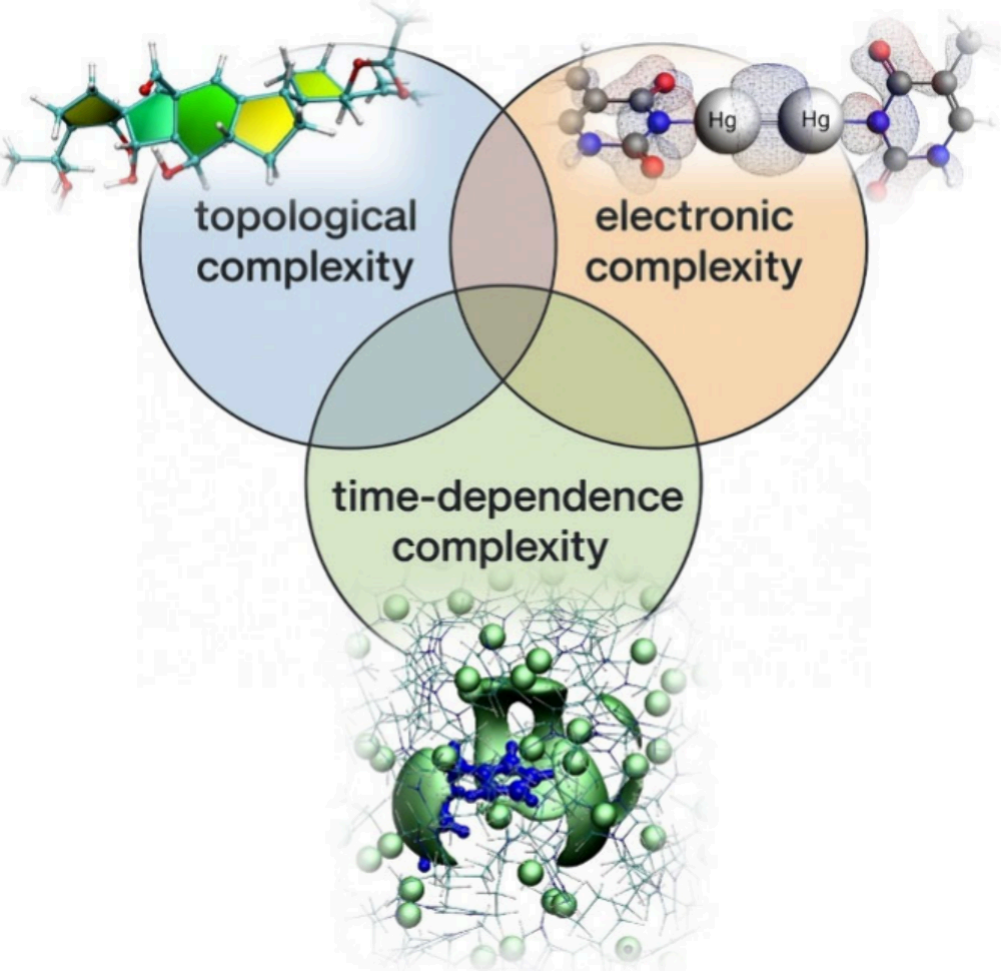
Complexity of an NMR spectrum–which ultimately is the complexity in the response of the electronic distribution around the nucleus of interest to external magnetic fields–may arise from three main sources. (i) It can occur through covalent bonds in a complex molecular topology, e.g., in natural products (vannusal B from ref 7); (ii) it can be directly encoded in the electronic structure through relativistic contributions, e.g., spin–orbit coupling (a thymine dimercury complex from ref 8); (iii) it may stem from a time dependence in the local environment affecting the solute electronic structure through noncovalent interactions (an imidazolium cation in the bulk phase of [C_4_C_1_im]Cl ionic liquid from ref 9). In all of these cases, the extensive knowledge base of empirical rules and relationships that allows us to extract structural and/or geometrical information from the spectra fails to drive the experimental chemist to the elucidation of the molecular structure. It is then useful to resort to computational NMR.

The first two cases are concerned with covalent molecules where the molecular structure is a well-defined concept: it is the fixed arrangement of the constituent atoms, as described either by their Cartesian or internal coordinates. Here, when the deduction of the structure from the NMR data is too challenging and the above information flow does not actually flow, computational NMR may provide a very useful contribution. The basic idea is to calculate the NMR parameters using some benchmarked DFT method for a set of putative structures and compare the calculated results of each of them with the experimental ones by means of several statistical parameters, such as Pearson’s correlation coefficient, Mean Absolute Error (MAE), etc. The agreement or lack thereof will allow one to retain or discard a given proposal. The structural space to be explored is therefore discrete, and it comprises all the molecules that can be envisaged based on an empirical interpretation of the experimental spectra. This approach was pioneered more than 25 years ago by Bagno,^10^ Bifulco,^11^ Köck,^12^ and Sebag^13^ and later named by Bagno as “reversing of the information flow”,^14^ since one starts from the geometry of a hypothetical molecule, calculates the NMR parameters by DFT (or possibly other *ab initio* QM methods), and compares them with the experimental ones (or simulate the proper NMR spectra). One key point that we should keep in mind is that, in order for this reverse flow of information to properly work, we must assume that the DFT-NMR calculation step is free from errors. This is of course not the case; DFT methods suffer from approximations both in the choice of the functional and in the selection of the basis set. Moreover, putative geometries need to be first energy minimized; therefore, the approximations can be found both at the geometry optimization level and at the NMR level. This is, however, an issue easily solved by performing a preliminary benchmark of the accuracy of the level of theory used in order to obtain a reliable linear correlation with a given confidence band. Generally speaking, the performance of several modern functionals^15^ and the existence of several dedicated basis sets for NMR calculations^16^ guarantee a very good performance of DFT methods; therefore, a disagreement between calculated and experimental data can be considered to stem only from the choice of a wrong geometry. This is the essential ingredient that allows us to use computational NMR as a tool to judge the reliability of a given structural proposal. These methods can be straightforwardly applied to covalent molecules, keeping in mind that if heavy atoms are the subject of investigation, relativistic versions of the DFT approach must be used.^17,18^ To give a hint about the accuracy of DFT-NMR methods, MAEs in proton and carbon chemical shifts of relatively rigid organic molecules, after inclusion of solvent and vibrational effects, were found to be <0.01 ppm for protons and <2 ppm for carbons, respectively.^19^ On the contrary, it is difficult to provide general statistical parameters measuring the performance of DFT protocols for cases involving heavy metals. This is because of the large and different chemical shift range and strength of the relativistic effects for each element of the periodic table.

The third case is qualitatively different. When the NMR properties are strongly influenced by the environment, the problem is still a structural problem, but the structure is then the average bulk structure of the phase. Average means that a time dependence needs to be considered. Bulk means that a relatively large number of molecules interacting through noncovalent interactions need to be accounted for. Typically, although the effect is limited to the first solvation shell of the reference nucleus,^20^ the dynamics of both the “solute” molecule (the one for which the NMR data are measured) and the “solvent” need to be taken into account. Therefore, contrary to the previous case, it is not possible to run a limited set of calculations for qualitatively different covalent structures. Instead, molecular dynamics (MD) is the only technique that can provide the set of coordinates of the system and its time dependence, in order to run the subsequent DFT-NMR calculations.

MD is, however, strongly limited by the accuracy of the force field (FF) parameters used. For simple liquids, the development of a FF follows well-established strategies, and more importantly, there are two experimental pillars that can be used as a reference to check the performance of the FF, that is, the density and the enthalpy of vaporization. If the simulation with a given FF provides an accurate estimate of the density, it means that the repulsive part of the intermolecular potential is probably correct. On the other hand, if the enthalpy of vaporization of the system is accurately reproduced, it is an indication that the attractive part of the intermolecular potential is likely correct.

However, for complex condensed phases, such experimental data might be missing. For example, the enthalpy of vaporization of ionic liquids or high-weight polymers is normally not available. Thus, the development of FF for these systems has to proceed, at least in part, blindly without the possibility of a double check. For example, at the beginning of 2000, several FFs for ionic liquids have been presented in the literature and, reasonably at that time, the sum of the partial charges of the cations and anions, was set to an integer value, e.g., +1*e* for imidazolium and −1*e* for halides, tetrafluoroborate, etc.^21^ However, after a few years, the community working in the field realized that such FFs were significantly wrong in predicting the dynamic properties, such as diffusion coefficients. The reason was traced back to the large polarizability and charge transfer effects present in ILs, which resulted in an effective charge lower than unity.^22^ One theoretically sound way to solve this problem was to develop polarizable FFs, which however still do not account properly for CT effects and hydrogen bonds.^23^ A simpler and computationally much more efficient way was to use scaled charges by a factor of about 0.8.^24^ Still, this approach is not fully satisfactory and novel FFs for ILs are continuing to be reported in the literature.^25^

Similarly, FF parameters for polymers, particularly polymers forming porous membranes, are normally “transferred” from simpler FFs developed for simpler liquids but without any warranty of transferability.^26^

Computational NMR can also play a role in these cases as a tool to test the validity of a given average bulk structure of a complex phase obtained from an MD simulation. Similarly to the case of covalent compounds, the comparison between calculated and experimental results is the key to gaining information about the goodness of the average bulk structure and therefore of the FF interaction parameters. However, in contrast to the previous case, the “structural space” is now continuous, since the intermolecular FF parameters, namely, the atomic Lennard-Jones contact distances and potential well depths, as well as the atomic partial charges, can be varied continuously within a certain range.

The extension of computational NMR to complex phases dominated by intermolecular interactions where the experimental chemical shift is actually the result of an average interaction of the solute molecule with its environment is a relatively recent development.^27,28^ The shift of paradigm is that MD simulations do not simply provide the background to evaluate the effect of the environment of the NMR, rather the comparison with experiments will be a reference to guide the changes and improvements in the FF field itself. It is the aim of this Account to report on recent applications from my own laboratory and some relevant literature of other research groups.

## 2 Covalent Systems

Concerning natural products, our laboratory was involved in computational NMR studies of strychnine, corianlactone, daphnipaxinin, and boletunone B,^29^ arsenicine,^30^ vannusal B,^7^ hexacyclinol,^31^ and halogenated marine products.^32^ The applications of computational NMR to natural substances has been, in recent years, significantly extended by Smith and Goodman^33,34^ and Sarotti and Zanardi^35–38^ who made impressive advances in this field by developing sophisticated methodologies for the comparison of calculated and experimental data. The interested reader is directed to the relevant literature cited above for a detailed account of these methods and to a recent review paper on the topic.^39^ It might be useful here to recall that after a level of theory has been selected the statistical parameters are obtained by correlating the calculated results of ^1^H and/or ^13^C (and possibly spin–spin coupling constants *J*) of each putative structure with the experimental data of the unknown compound. For natural products, these data are usually a relatively large set of numbers making the linear fit a reliable tool for the statistical analysis.

In contrast, for organometallic complexes with heavy atoms, the chemical shifts affected by relativistic effects are normally limited to the heavy atom itself or light atoms directly bonded to it (HALA, Heavy Atom on Light Atom effect^40^) even when the molecular structure is relatively simple. In such cases, it is convenient to run a benchmark using several known systems to build a proper correlation line and then use this correlation for the new unknown compound.

Over the years, several heavy nuclei have been investigated in our laboratory by computational NMR, including ^103^Rh,^41,42^ ^113^Cd,^43^ ^119^Sn,^44^ ^125^Te,^45^ ^129^Xe,^46,47^ ^195^Pt,^48,49^ and ^205^Tl,^50^ as well as the NMR of light nuclei bonded to heavy ones, e.g., iodinated marine natural products,^32^ iridium hydrides,^51^ and halobenzenes.^52^

The question we want to answer by applying computational NMR to covalent systems is: is this molecular structure right or wrong? I will highlight this approach with a recent example.

A thorough computational study concerning thallium NMR has been published.^50^ The experimental chemical shift of ^205^Tl spans a very wide range of about 7000 ppm. As shown in ref 50, relativistic effects are extremely large with the spin–orbit contribution (always shielding) varying from about 2600 ppm to about 11000 ppm. The paramagnetic term is also highly variable with the chemical structure, from about zero to −6000 ppm. It is therefore not possible to rationalize the observed trends in thallium compounds with simple qualitative empirical rules. It is also worth noting that for thallium the accepted reference for the chemical shift is an aqueous solution of TlNO_3_ at infinite dilution, which is extremely difficult to be modeled with a single DFT calculation. In such cases, it is better to correlate directly the calculated shielding constants with the experimental chemical shifts. This will give a correlation line with ideally a negative slope of −1 and an intercept representing the shielding constant of the reference compound; see eq 1. This is shown in Figure 2 for the set of benchmark compounds used in ref 50.

**Figure 2:**
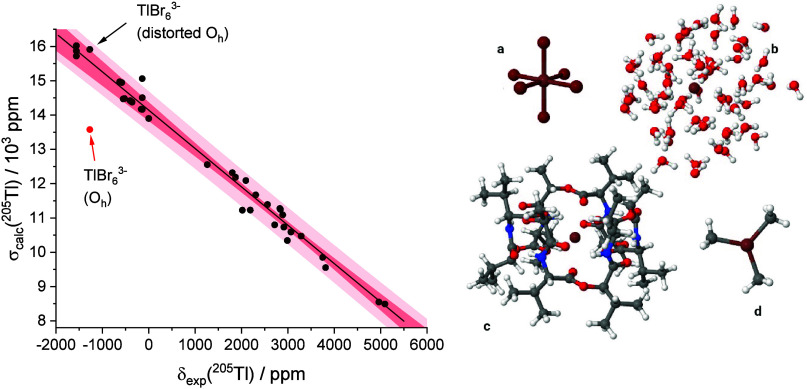
Left panel: Correlation between calculated shielding constants (NMR: ZSO-COSMO-PBE0/QZ4P­(Tl);DZP­(X). Geometry: ω-B97XD-PCM/LANL2DZ­(Tl);6-31+g*­(X)) of ^205^Tl and the experimental chemical shifts using several inorganic and organometallic systems described in ref 50. 95% confidence band for the fitting line (darker area) and prediction band (lighter area). The red dot data is the calculated shielding constant vs experimental chemical shift for Tl­(III)­Br_6_^3–^ using a symmetric octahedral geometry (*R*_Tl–Br_ = 2.58 Å) as determined in ref 53 by X-ray, while the black dot indicated by an arrow is the calculated result using the distorted octahedral geometry (three pairs of opposite bond lengths of *R*_Tl–Br_ = 2.83, 2.81, and 2.64 Å, respectively) in ref 54, which is in much better agreement. Right panel: some of the thallium compounds investigated in ref 50: (a) TlBr_6_^3–^; (b) the model Tl­(H_2_O)_60_^+^ of the NMR reference standard ^205^Tl, an aqueous solution of TlNO_3_ at infinite dilution; (c) the supramolecular complex Tl^+^@valinomycin; (d) Tl­(CH_3_)_3_, a covalent compound. Adapted with permission from Saielli, G. Computational NMR spectroscopy of ^205^Tl. *J. Comput. Chem.* **2023**, 44, 2016-2029, licensed under CC BY 4.0.

It appears that of all of the compounds investigated, encompassing several covalent organometallic and inorganic Tl derivatives and supramolecular complexes, one is clearly outside the prediction band of the correlation line (the red point in Figure 2). This corresponds to [Co­(NH_3_)_6_]­TlBr_6_, where, according to the X-ray data in ref 53, Tl­(III) has a perfectly symmetric octahedral geometry. However, other TlBr_6_^3–^ salts have been reported in the literature to have a distorted octahedral symmetry.^54^ Indeed, using such distorted geometry for the hexabromothallate anion, a much better agreement with the experiment is obtained; see the point indicated by an arrow in Figure 2. Thus, a comparison of calculated and experimental results suggests that the geometry of TlBr_6_^3–^ in [Co­(NH_3_)_6_]­TlBr_6_ is in fact a distorted octahedron. It is also noteworthy that the calculated chemical shift for the Tl­(I) aquaion obtained in ref 50 is very well reproduced using an energy minimized cluster of Tl^+^ surrounded by 60 water molecules. The calculated shielding constant, 13900 ppm, is only 200 ppm off the expected value obtained from the intercept (a relative error of just 1.4%). Several supramolecular complexes have also been investigated in ref 50, e.g., Tl@valinomycin among others (see Figure 2), and all show a very good correlation between the calculated shielding constant and the experimental chemical shifts. These results highlight the power of relativistic DFT calculations for the prediction of NMR properties of heavy atoms in both covalent and supramolecular complexes of organic and inorganic nature and, therefore, the usefulness of computational NMR as an aid in structural elucidation of organometallic compounds.

## 3 Bulk Phases

The effect of the chemical environment on the NMR properties can be observed for cases where the approximation of a relatively low-polar/poorly polarizable solute in a low-polar environment breaks down. This occurs for example for highly hydroxylated solutes in water, such as carbohydrates^55^ and polyols;^56^ for ionic liquids (ILs),^47,57^ which will be discussed at the end of this section; and for highly polarizable NMR probes like xenon.^58,59^ Using ^129^Xe chemical shift as a probe of the structure of the chemical environment is a well-established technique since Xe is highly polarizable (thus sensitive to the hosting matrix) and almost chemically inert.^60^ In fact, recent developments of ^129^Xe NMR have highlighted its potential as a probe of the environment not only using the chemical shift but also by means of “through-space” spin–spin *J* coupling.^61,62^ A recent “concept article” has been published on this topic,^63^ however, through-space spin–spin couplings will not be further discussed here.

An excellent work on the use of ^129^Xe NMR coupled with MD&DFT-NMR was published few years ago by Vaara and co-workers.^64^ The authors were able to obtain detailed information on the hydrophobic structure of water in the solvation shell of xenon by comparing calculated and experimental data. Moreover, the nonmonotonic trend of the chemical shift with the temperature was successfully rationalized. This work highlights the power of computational NMR to investigate the microscopic details of the structure of condensed phases.

We recently contributed to the field with two works concerning Xe@dihalomethanes^65^ and Xe@PIM (polymers of intrinsic microporosity).^66^ The chemical shift of xenon in the three dihalomethanes CH_2_Cl_2_, CH_2_Br_2_, and CH_2_I_2_, δ­(^129^Xe) at room temperature w.r.t. xenon gas, obtained by the group of Castiglione, is 189.9, 244.4, and 324.0 ppm, respectively.^65^ It is clear that it is strongly influenced by a noncovalent interaction with the solvent molecules. These can be of two types: steric interactions, related to the density and free volume accessible to xenon; van der Waals interactions, related to the large polarizability of both xenon and solvent molecules. The dependence of the xenon chemical shift on the density is well-known, and it has been extensively studied.^67^ The question we want to answer using computational NMR is what is the microscopic structure of the solvation shell of Xe in the three dihalomethanes and in which way it affects the xenon chemical shift? To rationalize the experimental observation, a combined MD&DFT-NMR protocol has been applied.^65^

First, a set of simulations was run with a FF derived from the LigParGen Web site^68^ and used “as is”. Then, clusters representing the Xe atom and its first solvation shell were extracted from the trajectory and used for the DFT-NMR calculations. The results were quite disappointing, and the disagreement was first traced back to a poor description of the density of the systems, especially for CH_2_I_2_. Therefore, we refined the FF parameters in order to enhance the agreement with the experimental densities, and this resulted in an improvement of the calculated chemical shifts of xenon in the three liquid phases.

The RDF of the distance between xenon and the dihalomethanes clearly showed that the interaction was preferably through the halogens, rather than the hydrogen atoms; see Figure 3a,b. To confirm the indications of the MD&DFT-NMR protocol, we also calculated the chemical shift in Xe···CH_2_X_2_ van der Waals pairs with different orientations; see Figure 3c. The DFT calculations on the model pairs agreed with the results of the MD and showed that the interaction of xenon facing the halogen atoms is capable of reproducing the observed trends in the chemical shifts, while the geometry with the CH_2_ moiety interacting with xenon does not. Thus, a careful comparison of experimental and calculated chemical shifts allowed us to infer detailed information on the microscopic structure of the solvation shell of xenon and validate the average bulk structure obtained by the refined FF.

**Figure 3:**
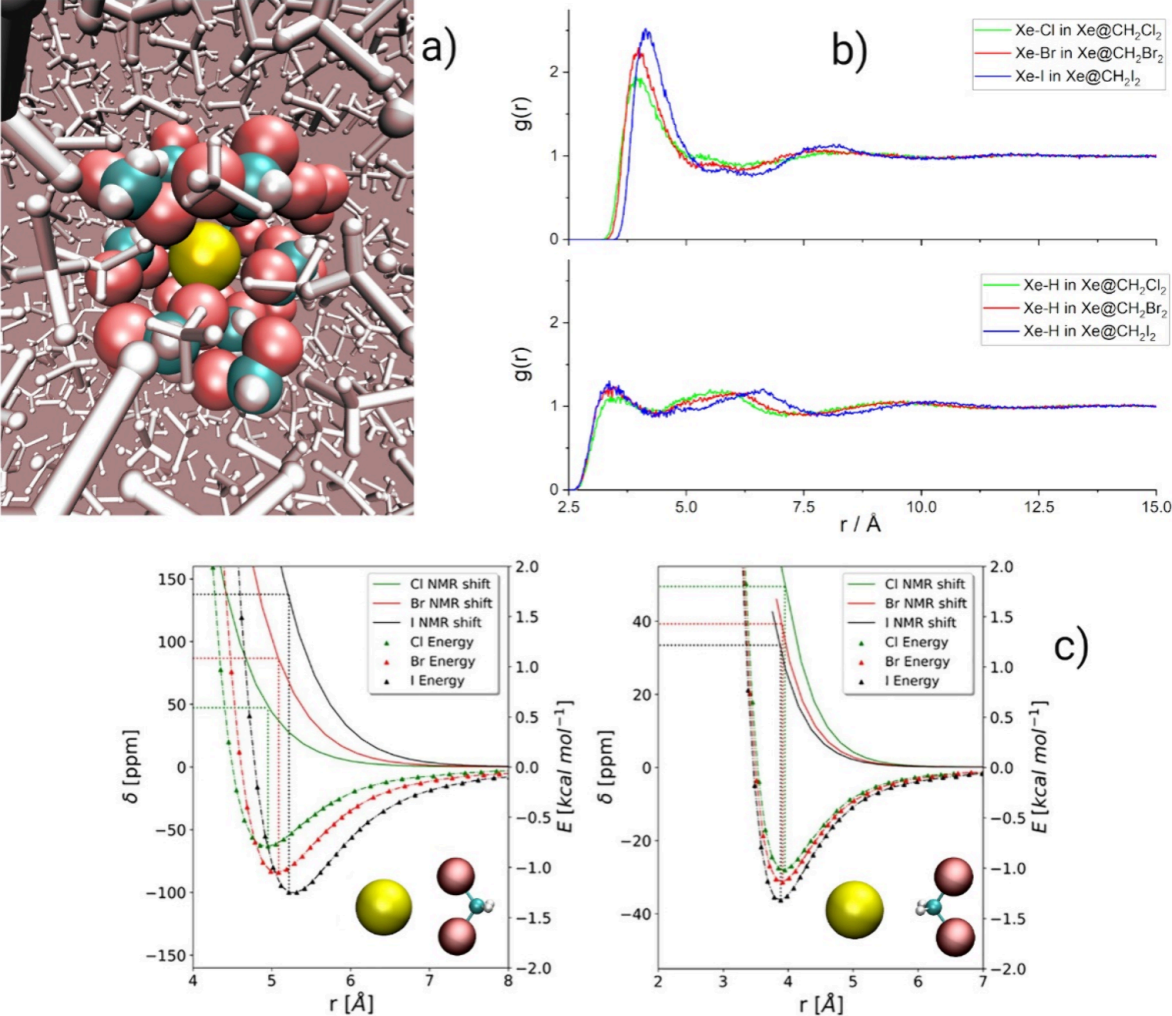
(a) Cluster of Xe@CH_2_Br_2_ obtained from the MD simulations clearly showing the preference of halide atoms (red) close to the xenon atom (yellow) rather than the H atoms (white). (b) RDF of the distance between xenon and the (top panel) halide atoms and (bottom panel) hydrogen atoms for Xe@CH_2_X_2_. It is clear that the probability of finding a halide atom in the first solvation shell is much greater than the probability of finding a hydrogen atom. (c) DFT calculated pair interaction energies (ZORA-SO-BLYP-G3/QZ4P level) and chemical shifts (ZORA-SO-BLYP/QZ4P­(Xe);TZ2P­(C,H,X)) of xenon for Xe···CH_2_X_2_ with a different relative orientation: on the left halide facing xenon and on the right hydrogen facing xenon. The geometry with the halides facing xenon reproduces the correct trend of the experimental chemical shifts. Adapted with permission from Boventi, M.; Mazzilli, V.; Simonutti, R.; Castiglione, F.; Saielli, G. Exploring the Structure of Halomethanes with Xenon: An NMR and MD Investigation. *J. Mol. Liq.* **2023**, 382, 122011, licensed under CC BY 4.0.

The second case is concerned with xenon in a membrane based on a polymer of intrinsic microporosity (PIM).^66^ The porous structure of PIM is the key structural feature behind their performance as membranes for separation technology.^69^ However, since they are amorphous, X-ray diffraction cannot be used to determine the structure, and a clear microscopic view can only be obtained by MD simulations. The question we want to answer here is what is the pore size distribution and how does it affect the xenon chemical shift? In ref 66, we investigated this problem with a MD&DFT-NMR protocol. As with the previous cases, MD trajectories of xenon dissolved in a PIM membrane based on ethanoanthracene units linked by methanodiazocene, produced by the group of Tocci,^69^ were used to generate a statistical ensemble of clusters containing xenon with the first solvation shell of the polymer; see Figure 4a.

**Figure 4:**
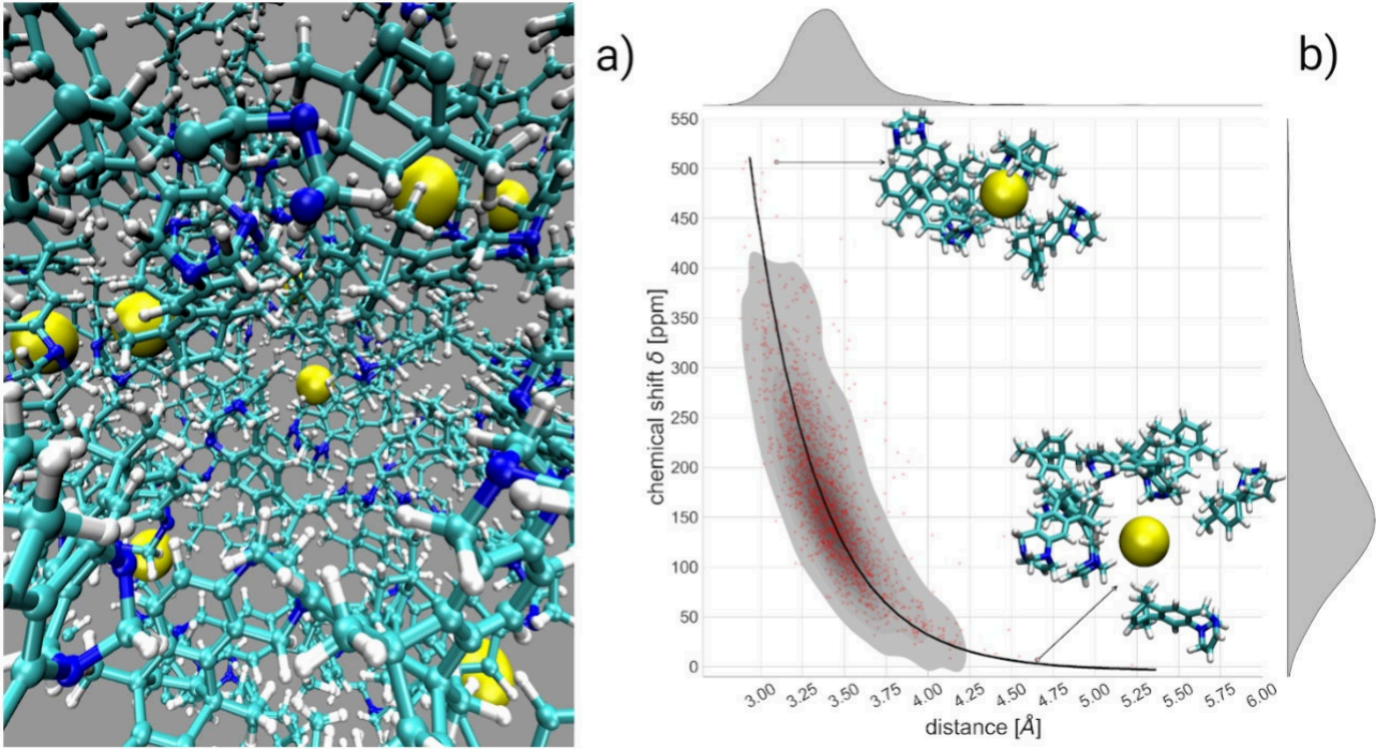
(a) Snapshot of the structure of the porous PIM membrane obtained from the MD simulation showing a large void and xenon atoms (yellow) dispersed within the matrix. (b) Calculated chemical shift for various clusters extracted from the MD trajectories. Two extreme cases are shown of a cluster with a very small pore hosting the Xe atom (high deshielding of ^129^Xe) and a cluster with a very large pore (low deshielding of ^129^Xe). The black line is the theoretical curve describing the dependence of δ­(^129^Xe) as a function of the distance from a linear alkane obtained in ref 70. The curve perfectly models the Xe@PIM chemical shift dependence on the distance from the inner pore walls. On the top and right axis, the projected distributions of Xe-inner pore wall distances and chemical shifts δ­(^129^Xe), respectively. Adapted with permission from Mazzilli, V.; Rizzuto, C.; Tocci, E.; Saielli, G. ^129^Xe NuclearMagnetic Resonance in Polymeric Membranes: A Computational Study of the Effect of Pore Size and Void Distribution on the Xenon Chemical Shift. *J. Phys. Chem. B* **2025**, 129 (42), 11090, licensed under CC BY 4.0.

The subsequent DFT calculations allowed us to obtain a calculated chemical shift which, when referenced to xenon in *n*-hexane, showed a very good quantitative agreement with the experimental results.^69^ Most importantly, the dependence of the calculated chemical shift from the inner walls of the pores could be nicely modeled using a theoretical exponential dependence derived several years ago using model systems made by a single Xe atom interacting with a linear alkane as a model of polymeric organic membranes.^70^ This is shown in Figure 4b. As expected, when the Xe atom is hosted in narrow pores, the deshielding is very large because of steric interaction. It is, however, remarkable that the dependence of δ­(^129^Xe) on the distance from the organic moieties seems to follow a general law with an exponential decay constant of 0.47 Å^–1^.

The last case is concerned with the proton chemical shifts, δ­(^1^H), of imidazolium cations in pure ILs with different anions.^9^ Due to the strong electrostatic interactions, the chemical shift of the imidazolium ring protons, especially H2, the hydrogen atom bonded to the carbon between the two nitrogens of the ring, is strongly dependent on the counteranion.^28^ For example, in 1-butyl-3-methylimidazolium, the H2 resonance varies from 8.53 ppm in neat [C_4_C_1_im]­[BF_4_][Bibr ref71] to 10.23 ppm in neat [C_4_C_1_im]­Cl.^72,73^ Such an environmental effect resulting from noncovalent interactions, Δδ = 1.7 ppm, is extremely large and of the same order as inductive and mesomeric effects due to covalent bonding in organic molecules. Moreover, the striking feature is the very large deshielding of H2 in the chloride salt, related to the strong C2–H2···Cl hydrogen bond. Thus, we endeavor to understand and reproduce the chemical shift of two ILs with [C_4_C_1_im] cation and chloride and tetrafluoroborate as counteranions by means of a combined MD&DFT-NMR computational protocol. The questions we want to answer using computational NMR are: what is the local average structure of the chloride anions around the imidazolium ring, in particular around H2, and how does it affect the proton resonance?

A recent paper by Aidas and co-workers successfully modeled the chemical shift dependence of the butylimidazolium cation in [C_4_C_1_im]­[BF_4_] as a pure salt and in a mixture with water^74^ by means of a combined MD&DFT-NMR protocol. However, in another paper concerning [C_4_C_1_im]­Cl, they stated that “*Our results thus suggest that a refinement of the force field used in the present MD simulations of neat IL may be necessary in order to improve the local distribution of ions around the C2–H2 moiety of imidazolium cations*”.^72^ Clearly, the hard chloride anion is a rather difficult case to be modeled with classical FFs since its interaction with the positively charged H2 imidazolium ring proton stems not only from electrostatic contribution but also from a subtle combination of polarizability and charge-transfer effects. This would require a QM treatment of the PES for a correct description of the dynamics, but on the other hand, QM MD simulations cannot explore the long times and large boxes necessary for such viscous and nanosegregated fluid systems.

To gain insights on this issue, in ref 9, we investigated four different classical FFs for [C_4_C_1_im]­[BF_4_] and [C_4_C_1_im]­Cl: a full-charge FF, a scaled-charge FF, a polarizable FF, and a recently presented FF with virtual charges;^25^ all MD runs were performed by the group of Wang.^9^ For each trajectory, we extracted 100 configurations of clusters made by a central butylmethylimidazlium cation and its solvation shell (with different cutoff radii) and ran DFT-NMR calculations following several protocols detailed in ref 9. The main result is summarized in Figure 5 where we also add the results obtained some years ago for a similar imidazolium chloride IL ([C_2_C_1_im]­Cl) using a Car–Parrinello MD to generate the trajectory (though over a relatively short time scale).^75^

**Figure 5:**
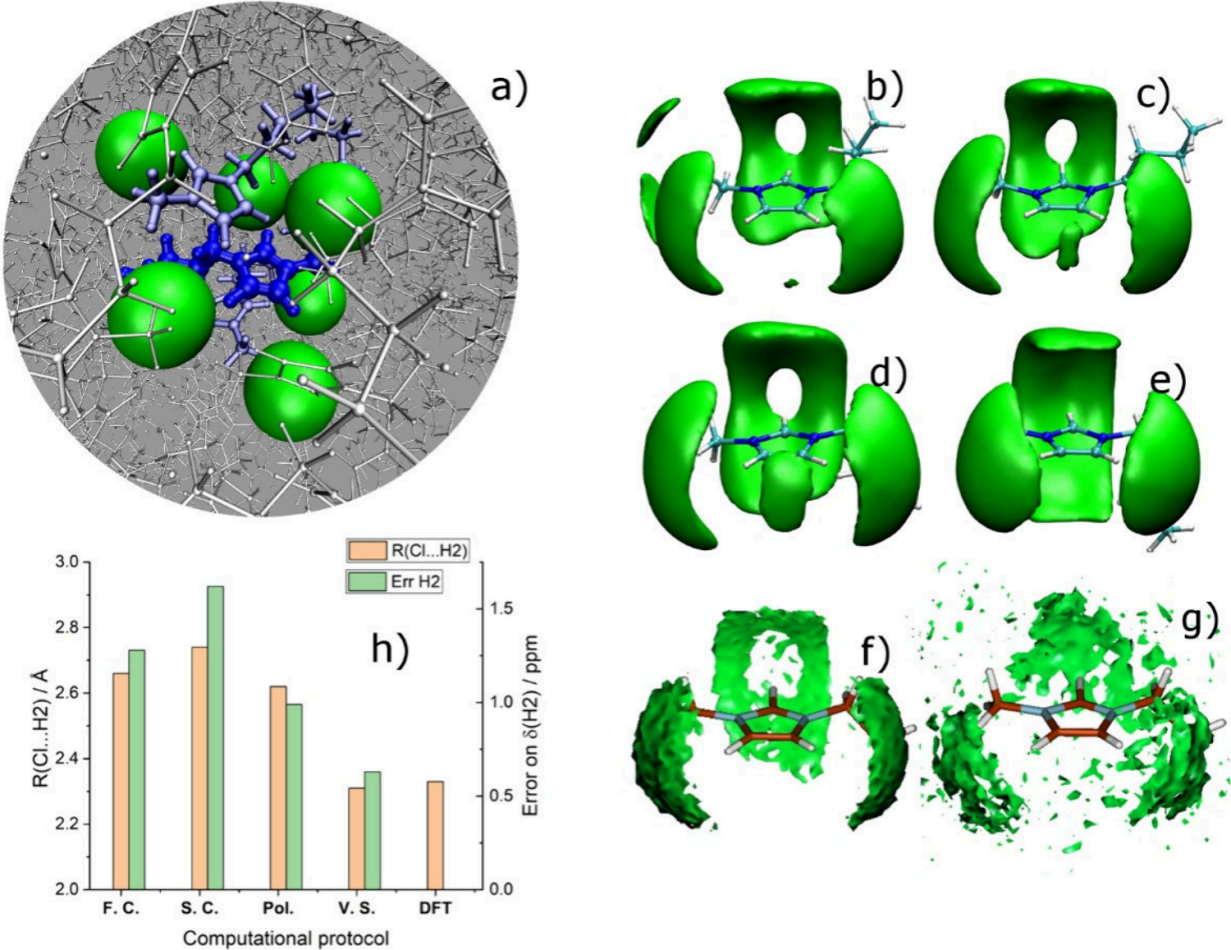
(a) Example of a cluster of [C_4_C_1_im]Cl extracted from the MD trajectory and submitted for DFT-NMR calculations. The reference cation for which the chemical shift is sought is in dark blue at the center; three other cations (light blue) and six chloride anions (green) are part of the first solvation shell of the reference cation. (b–e) Spatial Density Functions (SDFs) of the probability to find a chloride anion around the imidazolium ring: (b) full-charge classical FF; (c) scaled-charge classical FF; (d) polarizable classical FF; (e) virtual-site classical FF. (f, g) SDFs of [C_2_C_1_im]­Cl: (f) classical full-charge FF; (g) Car–Parrinello MD. (h) Cl···H2 distance of minimum energy using various classical FF for an ideal ion-pair plus the value obtained by DFT, and the error on the calculated proton chemical shift of H2 in [C_4_C_1_im]­Cl, using MD&DFT-NMR protocols with the same FFs: the two properties follow an almost identical trend. (a)−(e) Adapted with permission from Zhu, R.; Yan, T.; Wang, Y.; Saielli, G. Towards Quantitative Prediction of Proton Chemical Shifts in Imidazolium Chloride Ionic Liquids by Computational NMR. *Phys. Chem. Chem. Phys.* **2025**, 27 (47), 25310−25321, licensed under CC BY 4.0. (f)-(g) Adapted with permission from Bagno, A.; D’Amico, F.; Saielli, G. Computing the 1H NMR Spectrum of a Bulk Ionic Liquid from Snapshots of Car-Parrinello Molecular Dynamics Simulations. *ChemPhysChem* **2007**, 8 (6), 873−881. Copyright Wiley-VCH, 2007.

The results shown in Figure 5 clearly highlight the reason an accurate modeling of the ring proton chemical shift, particularly H2, is difficult: classical FFs generally fail in producing a correct average geometry of chloride around the hydrogen atoms. From a qualitative point of view, the SDFs obtained with relatively old FF (panels b–d) all exhibit a “hole” of probability, that is a local minimum, for the chloride pointing almost collinear with the C2–H2 bond. In contrast, the more recent virtual-site FF does not. This result is qualitatively in agreement with the ones obtained some years ago using Car–Parrinello MD of a very similar salt, [C_2_C_1_im]­Cl, where the classical FF exhibited the “hole” in the SDF while the CPMD SDF did not.^75,76^ This suggests the presence of an energy barrier between two tilted orientations of the chloride anion, in the plane of the imidazolium ring, corresponding to two minima on the two sides of the C2–H2 bond, respectively; see the detailed discussion in ref 9. Such an energy barrier is relatively high for the first three classical FFs, while the path is almost barrierless for the virtual site FF and the DFT based MD trajectory. On the other hand, the distance of approach of the anion to the H2 atom is also quite different: in Figure 5h, we show the trend of the minimum energy distance calculated with the four classical FFs and with DFT, paired with the error on the proton chemical shift of H2 obtained from the MD&DFT-NMR protocols. It is clear that the error closely follows the trend of the distance. Therefore, this is, together with the energy barrier, the key parameter that should be addressed in a revised version of classical FFs for MD simulations of the imidazolium halide system. We should mention that, while it is relatively clear which are the structural features responsible for an improved performance of a given FF (here, the minimum distance of approach and the energy barrier), it is less straightforward to ascribe such features to a specific kind of interaction (LJ contact distance, LJ well-depth, partial charges, etc.), or some combination of them, a work that requires additional efforts and thorough testing.

## 4 Conclusion

The process to validate a given structural proposal by a comparison of calculated and experimental NMR properties exhibits a marked difference for covalent molecules and bulk phases. In the first case, the identity of the molecule, that is, the topological arrangement of the atoms, is normally the unknown feature that at some point has to be “guessed”, though guided by NMR data. Several putative structures, representing the “discrete structural space” mentioned in the Introduction, can be conceived and put to the test. The exact geometrical parameters (bond lengths and bond angles) do not represent a problem since they can be easily obtained for each structure by energy minimization using appropriate DFT methods.

In contrast, for bulk phases, the qualitative structure is more or less clear: almost all available FFs provide similar solvation shells for hydrophobic probes in polar solvents; they all predict the microscopic structure of ionic liquids with alternation of cationic and anionic layers and long-range nanosegregation between ionic and akylic moieties.^77,78^ The same can be said concerning the porous structure of polymeric membranes. What is not known in these cases, where the structure comes from *empirical* classical FFs, is the exact average geometrical arrangement of the bulk phase. This is the “continuum structural space” to be investigated, as mentioned in the Introduction, and useful information can be obtained from the comparison of calculated and experimental NMR data that can be used as a guide to tune new FFs with improved performance.

This difference is schematically represented in Figure 6. The potential energy surface on the left represents some of the many possible isomers of a given molecule, the structure of which is sought. When analyzing the NMR spectra of a newly isolated unknown molecule, we may not be able to select the right potential well, that is, infer its identity. In contrast, the free energy surface on the right, schematically representing the free energy minimum of a bulk phase structure as obtained from MD simulations, may be qualitatively correct, but we likely do not have an accurate quantitative description of the average geometrical parameters due to shortcomings in the FFs. In both cases, resorting to computational NMR will provide a useful litmus test to judge the correctness of the structural/geometrical proposal.

**Figure 6:**
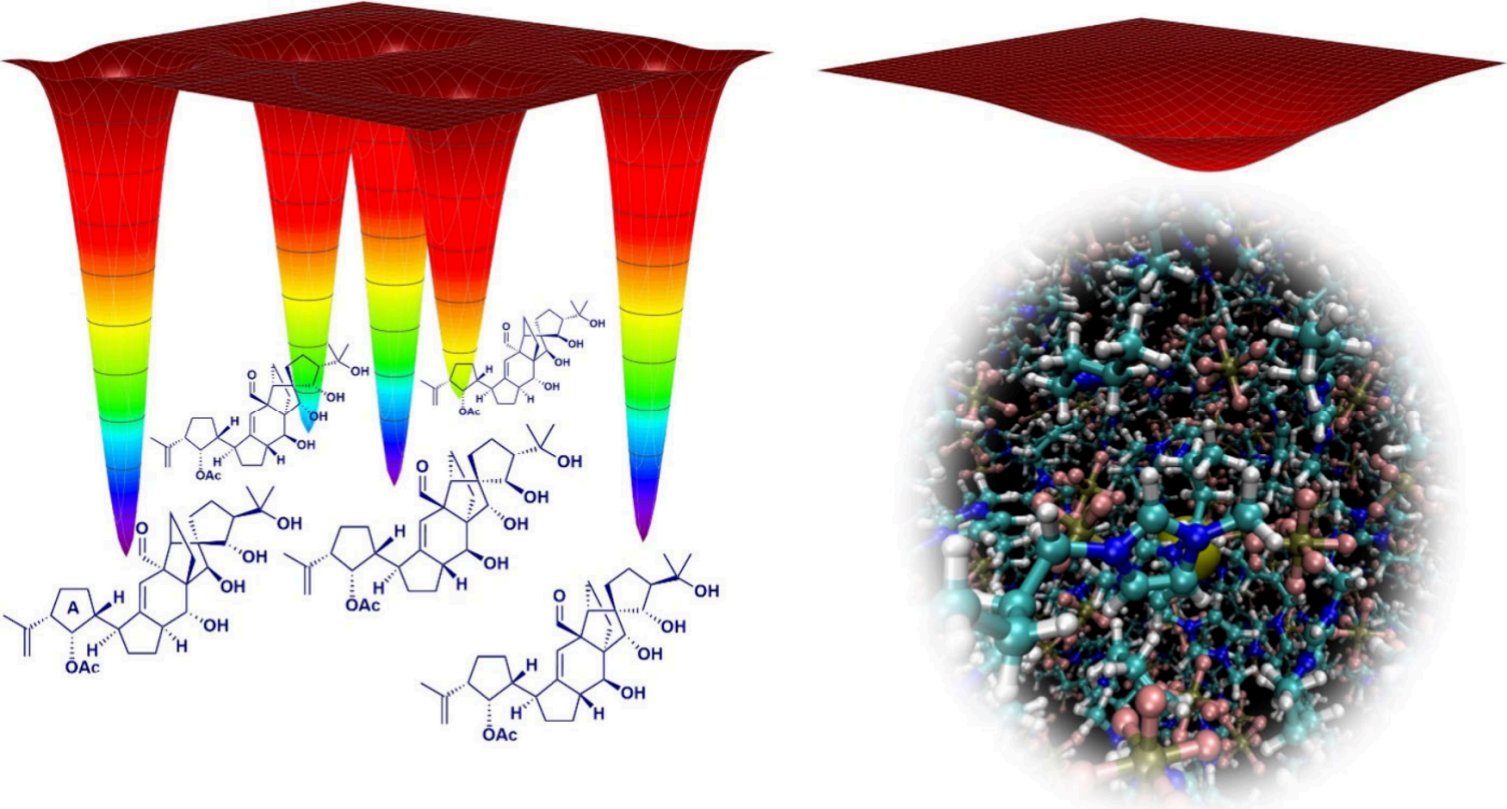
For covalent systems, the structural space to be explored is discrete: there is a number of conceivable molecular structures, each one represented by a well-defined energy minimum (Left panel: some of the vannusal B stereoisomers investigated by computational NMR in ref 7). The unknown variable here is which is the correct energy well, meaning the correct molecular structure which is responsible for a given NMR spectrum. Comparison of the calculated and experimental NMR data will help to solve the puzzle. On the other hand, in bulk phases, the structural space to be explored is continuous (Right panel: solvation shell of xenon in imidazolium ionic liquids investigated in ref 47). The noncovalent time-averaged bulk geometry is normally qualitatively known; however, the exact geometrical details are subject to severe indeterminations due to the many approximations of the classical force fields used. Here, the comparison of calculated and experimental NMR data will allow us to test and adjust the FF parameters, thus advancing toward a more realistic description of the bulk phase structure of the condensed phase.

I conclude by mentioning a type of structural complexity that has not been considered here and which represents a challenging endeavor for both experimental and computational NMR, namely, paramagnetic systems. Despite NMR being long regarded as a technique for closed-shell molecules, it is highly informative also for systems with unpaired electrons (such as organic radicals and organometallic compounds).^79^ Computational NMR is also providing support for such cases,^80–84^ although accurately accounting for the large electron correlation effects represents an arduous task for modern quantum chemical methods.

## Abbreviations

FF: force field
NMR: nuclear magnetic resonance
DFT: density functional theory
MD: molecular dynamics
FID: free induction decay
QM: quantum mechanics
PIM: polymer of intrinsic microporosity
LJ: Lennard-Jones
